# Mental Resilience and Coping With Stress: A Comprehensive, Multi-level Model of Cognitive Processing, Decision Making, and Behavior

**DOI:** 10.3389/fnbeh.2021.719674

**Published:** 2021-08-06

**Authors:** Iryna S. Palamarchuk, Tracy Vaillancourt

**Affiliations:** ^1^Counselling Psychology, Faculty of Education, University of Ottawa, Ottawa, ON, Canada; ^2^School of Psychology, Faculty of Social Sciences, University of Ottawa, Ottawa, ON, Canada

**Keywords:** cognitive appraisal, cerebral functional activity, coping, decision making, executive functioning, psychological stress

## Abstract

Aversive events can evoke strong emotions that trigger cerebral neuroactivity to facilitate behavioral and cognitive shifts to secure physiological stability. However, upon intense and/or chronic exposure to such events, the neural coping processes can be maladaptive and disrupt mental well-being. This maladaptation denotes a pivotal point when psychological stress occurs, which can trigger subconscious, “automatic” neuroreactivity as a defence mechanism to protect the individual from potential danger including overwhelming unpleasant feelings and disturbing or threatening thoughts.The outcomes of maladaptive neural activity are cognitive dysfunctions such as altered memory, decision making, and behavior that impose a risk for mental disorders. Although the neurocognitive phenomena associated with psychological stress are well documented, the complex neural activity and pathways related to stressor detection and stress coping have not been outlined in detail. Accordingly, we define acute and chronic stress-induced pathways, phases, and stages in relation to novel/unpredicted, uncontrollable, and ambiguous stressors. We offer a comprehensive model of the stress-induced alterations associated with multifaceted pathophysiology related to cognitive appraisal and executive functioning in stress.

## Introduction

The impact of minor and major stressors on psychological and physical health is well documented. It is clear from this literature that stressors are salient stimuli, including events and behavior, that can evoke strong negative emotions and feelings such as fear, betrayal, confusion, and powerlessness (i.e., psychological stress), which in turn, can lead to significant morbidity including depression, PTSD, coronary heart disease, and ischemic stroke (e.g., Stansfeld and Candy, 2006; Hamer et al., 2012; Richardson et al., 2012; Brainin and Dachenhausen, 2013; Henderson et al., 2013; Wei et al., 2014a). Psychological stress is an appropriately evoked biological reaction intended to recalibrate and optimize executive functions to stay focused on the stressor at hand, and thus mitigate the potential harm to the organism. Although this mechanism is intended to be adaptive, it is not perfect, particularly in the case of intense and/or chronic stress. In this context, the neuroactivity can constrain cognition and increase the risk of mental and social dysfunction, as well as neural and systemic inflammation (e.g., Shin and Handwerger, 2009; Hassija et al., 2012; Latack et al., 2017; Auxéméry, 2018; Mills et al., 2019; Quinones et al., 2020; Slavich, 2020; Vaillancourt and Palamarchuk, 2021). The origin of this type of stress-associated cognitive maladjustment belongs to attentional tunneling (i.e., stressor preoccupation, e.g., Chajut and Algom, 2003; Roelofs et al., 2007; Pilgrim et al., 2010; Tsumura and Shimada, 2012; Shields et al., 2019), which restricts cognitive flexibility (e.g., Alexander et al., 2007; Shields et al., 2016; Marko and Riečanský, 2018), and distorts memory because aversive information is prioritized over neutral or positive information (e.g., de Quervain et al., 2009, 2017; Palamarchuk and Vaillancourt, under review; Vaillancourt and Palamarchuk, 2021). Moreover, despite the shift in cognitive defence mechanism to liberate the emotional burden *via* the downplaying of aversive feelings and thoughts, the attempted suppression of the stressor’s influence can still affect mental health. For instance, internalizing can lead to dysphoria or anhedonia (Salmon and Bryant, 2002), core symptoms of major depressive disorder (American Psychiatric Association, 2013).

**Figure 1:**
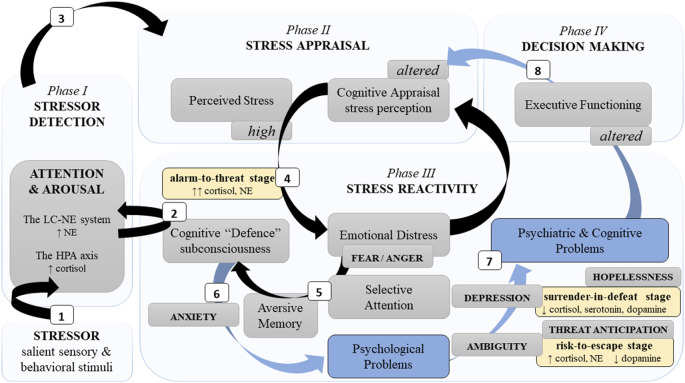
A simplified schema of the neurocognitive reactivity to a psychological stressor. Note. This schema presents major neurocognitive dynamics during stress development phases (light blue blocks) and stages (yellow blocks). Neurocognitive stress reactivity is facilitated by two principal neural limbs, the LC-NE system and the HPA axis. *Phase I*: (1) The LC-NE system detects a challenging stimulus (i.e., stressor) and “informs” the neocortex related to cognition. (2) Automatically, it triggers subconscious cognitive defence mechanisms to activate the HPA axis. *Phase II*: (3) Further engagement of cognitive appraisal defines the severity of a stressor. *Phase III*: (4) Severe stress perception distresses emotions. (5) Fear promotes selective attention and aversive memory which aggravates cognitive defence and (6) can result in psychological problems. (7) Insufficient fear downregulation in chronic and/or intense stress (alarm-to-threat stage), as well as chronic uncertainty (risk-to-escape stage) and/or losing hope (surrender-in-defeat stage) can lead to psychiatric disorders and cognitive alterations, e.g., poor memory and executive dysfunctions. *Phase IV*: (8) Consequently, poor neurocognitive functioning affects decision-making, as well as alters recognition (phase I), appraisal (phase II), and response (phase III) of/to a novel stressor. *Legend*: HPA—hypothalamic “pituitary” adrenal; LC-NE—locus coeruleus-norepinephrine; ↑: hyperactivity/increase; ↓: decrease; black arrows—adaptive path; blue arrows and blocks—maladaptive path.

The effect of a psychological stressor is primarily related to the level of perceived stress severity, i.e., cognitive appraisal/interpretation of the stressor. Stressors can represent various aversive events regardless of their proximity (i.e., direct or remote such as in witnessing or learning), which commonly disrupt emotional integrity (Figure 1). This mechanism and development have not been described comprehensively in one integrated model. In this review, we outline the central neural dynamics and highlight the main phases of stress development. We define a neuropathophysiological mechanism of psychological stress that represents a complex cognitive construct beyond the classic fear-conditioning model. We detail neural dynamics in stress, and in doing so, propose a multi-level model to describe the accumulated neuronal alteration of cognitive dysfunctions. Our review highlights the importance of ameliorating psychological assessment, clinical screening, prevention, and treatment of altered adaptive-learning abilities of psychologically distressed and depressed individuals.

## Stressor Detection and Arousal

**Figure 2:**
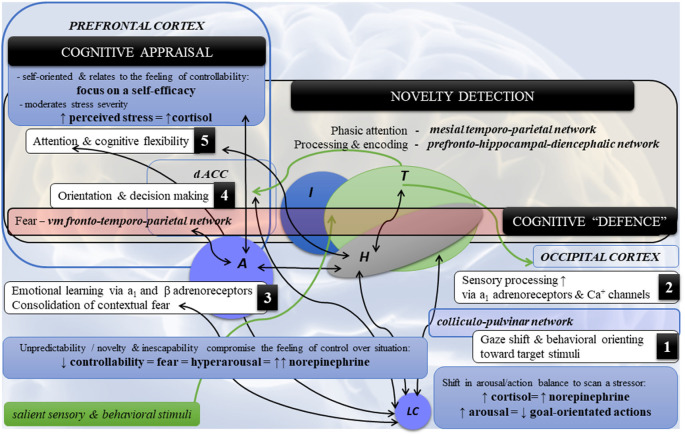
Highlights of the neural dynamics and topology in neurocognitive stress reactivity. Note. Schematic diagram of the main co-occurrences (1–5) in neurocognitive reactivity and cerebral topology in psychological stress. (1) Detection of a threat by the LC-NE system and (2) its sensory processing triggers (3) the amygdala (fear), which in turn affects (4,5) cognition and behavior *via* the ventromedial fronto-temporoparietal network [cognitive defence] directed towards fearful stimuli (the fronto-temporal circuits) and novel/unexpected stimuli (the fronto-parietal circuits). Novelty detection encompasses the following circuits: (a) *mesial* *temporoparietal network* for phasic attention to the novel stimuli such as auditory and somatosensory, but to the lesser degree visual; (b) *the prefrontal-hippocampal-diencephalic* *network* (i.e., frontocentral hippocampal regions, adjacent fusiform, lingual gyri, fornix-mammilothalamic-cortical pathways and calcarine) for novelty processing and encoding. By contrast, the posterior hippocampal region is associated with spatial processing and encoding. *Legend*: A—amygdala; dACC—dorsal anterior cingulate cortex; H—hippocampus; I—insula; LC—locus coeruleus; NE—norepinephrine; T—thalamus; vm—ventromedial; ↑: hyperactivity/increase; ↓: decrease; ↔: functional coactivity.

Psychological stress is a challenge, but the nervous system stands its homeostatic ground. First, it facilitates the detection of a stressor with noradrenergic signaling *via* the locus coeruleus-norepinephrine (LC-NE) system (e.g., Sara and Bouret, 2012; Bari et al., 2020; Poe et al., 2020). The LC-NE system is formed by the LC in the brainstem, which is a cluster of neurons encompassing NE. The axons of the LC neurons are organized in the several modules that project across the brain and format a noradrenergic system with extensive collateralization. Thus, LC activation results in a diffuse NE surge in the cerebral networks (e.g., Sara and Bouret, 2012; Szabadi, 2013; Schwarz et al., 2015; Bari et al., 2020; Poe et al., 2020), which is linked to cognitive (e.g., attention and flexibility) and behavioral outcomes (e.g., Skosnik et al., 2000; Morilak et al., 2005; Alexander et al., 2007; Figure 2).

The LC neurons can be subconsciously activated in response to fear, which is likely linked to the corticotropin-releasing factor (CRF) afferents from the amygdala (e.g., Pacak et al., 1995; Dunn et al., 2004; Valentino and Van Bockstaele, 2008; Sara and Bouret, 2012; Szabadi, 2013; Godoy et al., 2018; Reyes et al., 2019). The amygdala is principally associated with a fear response (e.g., Etkin and Wager, 2007; Godoy et al., 2018; Palamarchuk and Vaillancourt, under review). Chronic psychological stress strengthens the functional connectivity between the LC and amygdala that relates to fear learning. Specifically, *via* hypothalamic orexin, LC activity facilitates amygdala-dependent aversive/fear memory (e.g., Sears et al., 2013), with early retrieval (up to 6 h) associated with activated prelimbic prefrontal cortex (PFC) → basolateral amygdala circuits and later retrieval (up to 28 days) associated with activated prelimbic PFC → thalamic paraventricular nucleus → central amygdala circuits (rat model, Do-Monte et al., 2015). At the same time, prolonged severe stress has been found to impair amygdalar inhibition, seen in reduced PFC → basolateral amygdala connectivity that hyperactivated the amygdala and ensued aggressive behavior (Wei et al., 2018). That is, in chronic stress, the amygdala is relaxed from the PFC, yet thalamic pathways reconnect the pair, at least for fear memory retrieval.

The LC-amygdala connectivity is reciprocal as the amygdala can phasically activate LC neurons as well (e.g., Bouret et al., 2003). Liddell et al. (2005) showed that subliminal fear stimuli (i.e., fearful faces) coactivate the LC, amygdala, pulvinar, and frontotemporal areas related to orienting an “alarm system” (hereafter referred to as *cognitive defence* that is induced by “alarmed” LC-NE system; see Figure 2). Leuchs et al. (2017) validated previous findings that phasic pupil dilations, which are related to the LC activity (e.g., Murphy et al., 2014) in response to aversive (e.g., Wiemer et al., 2014) and emotionally arousing stimuli (e.g., Bradley et al., 2008), are a physiological marker of fear learning/conditioning. Fear learning is associated with a functional coactivity between the amygdala, anterior cingulate cortex (ACC), insula, thalamus, and PFC (e.g., Etkin and Wager, 2007; Fullana et al., 2016; see Figure 2. At the same time, almost all of the neocortex (e.g., the PFC related to cognitive appraisal and stress controllability; and the ACC together with the insula related to social monitoring/pain network; Palamarchuk and Vaillancourt, under review) can modulate LC activity *via* passing already processed/encoded information about the salient sensory and behavioral stimuli (e.g., Sara and Bouret, 2012; Szabadi, 2013; Schwarz et al., 2015).

The LC neuronal activity is a bimodal—tonic (sensory-orientated) and phasic (action-orientated)—firing that regulates attention and ongoing behavior. Specifically, the levels of tonic activity relate to drowsiness and disengagement (low), arousal (moderate), and hyperarousal (high; Sara and Bouret, 2012; Hofmeister and Sterpenich, 2015; Bari et al., 2020). Hyperarousal has been found to be associated with an increased effort to face challenges (Varazzani et al., 2015). The phasic activity increases in response to relevant behavior and hence prioritizes a goal-directed attentional processing over a stimulus-driven attention, which serves adaptive behavioral performance (Sara and Bouret, 2012; Hofmeister and Sterpenich, 2015). The phasic activity also reacts to fear, nociception (e.g., Valentino and Van Bockstaele, 2008; Sara and Bouret, 2012), and motivation (i.e., anticipated reward size; Bouret and Richmond, 2015), that modulate behavioral performance. However, upon detecting a stressor, the LC drops its phasic activity and increases its tonic activity, which is seen in hyperarousal and hypersensitivity and relates to scanning attention and the analysis of behavior (Valentino and Van Bockstaele, 2008). That is, when facing a stressor, the LC puts goal-directed attentional processing (the dorsal frontoparietal network) on hold so the challenge can first be inspected (the ventral/mesial frontoparietal network, mainly the dextral part including the inferior frontal gyrus, frontal/insula regions, and basal ganglia; Corbetta and Shulman, 2002; Corbetta et al., 2008; Shulman et al., 2009; see also Godoy et al., 2018). Therefore, we define cognitive defence as the ventromedial fronto-temporo-parietal network driven by fear which can emerge when fearful stimuli (frontotemporal circuits) and novel/unexpected stimuli (frontoparietal circuits; Figure 2) are presented.

Unexpected novel stimuli that do not have predictive value will elicit larger event-related potential responses measured by electroencephalography and prolonged reaction time to the subsequent target (i.e., larger arousal), that in turn, will modulate behavior (Knight and Nakada, 1998). The findings in shocked rats are that, compared to expected stressors, unpredictable stressors evoke greater LC-NE reactivity seen in the higher levels of principal NE metabolite in the amygdala, hypothalamus, and thalamus, and higher levels of corticosterone in plasma. In contrast, predictable stressors do not elevate NE metabolite levels in the LC and thalamus, nor corticosterone levels in plasma, the way unpredictable stressors do, compared to non-shocked rats (Tsuda et al., 1989). The potential mechanism of the higher impact of unpredictable stress may relate to altered serotoninergic (5-HT) signaling that relates to preserve the β-adrenoreceptors’ upregulation (e.g., Asakura et al., 2000; Yalcin et al., 2008), which is also seen in conditioned fear and inescapable stress (Kaehler et al., 2000). However, McDevitt et al. (2009) showed that although stress controllability modulates NE levels, it does not affect NE signaling in the LC neurons; whereas stressor controllability relates to the medial PFC function to downregulate the amygdalar hyperactivity associated with altered 5-HT signaling (e.g., Amat et al., 2005; see also Puig and Gulledge, 2011; Leiser et al., 2015; Garcia-Garcia et al., 2017; Palamarchuk and Vaillancourt, under review). The findings collectively highlight that neurocognitive stress reactivity is orchestrated by the LC-NE system, fueled by the fear-driven amygdala, and regulated by the PFC/5-HT circuits.

## Cognitive Appraisal of Stress Severity

Elevation of cortisol levels in response to a stressor is associated with perceived stress severity (e.g., Sladek et al., 2016; Gabrys et al., 2018, 2019; Woody et al., 2018). That is, a psychological threat “exists” to the extend cognition “sees” it. Though cognitive capability may help with the avoiding of dangerous situations, it is the cognitive appraisal that helps reduce psychological stress *via* a self-appraisal perspective that conquers challenges, but not the challenging stimulus *per se*. Slattery et al. (2013) tested the associations between three neurocognitive variables, IQ, academic achievement, and verbal/visual short–term memory, which were measured at age 14, during a standardized psychosocial stress paradigm delivered at age 18. Results indicated that poor cognitive appraisal, but not cognitive skill, predicted stress responses. Specifically, stress-coping abilities during stress anticipation depended on “secondary” cognitive appraisal related to the perception of poor self-efficacy (we term this appraisal related to the perception of self-efficacy to deal with the stressor *self-appraisal*), but not on “primary” cognitive appraisal (greater threat/challenge-perception, which we term *stressor-appraisal*). Poor self-appraisal independently predicted lower cortisol reactivity during the test indicating an insufficient stress response in adolescents. At the same time, poor visual memory predicted cortisol hyperreactivity to stress, whereas internalizing disorders increased the links between verbal memory and cortisol reactivity. These results denote an important fact that intelligence alone is not likely a marker of emotion regulation that is sufficiently related to stress outcome. Rather, the outcome associated with stress is principally influenced by an individual’s cognitive self-appraisal.

**Figure 3:**
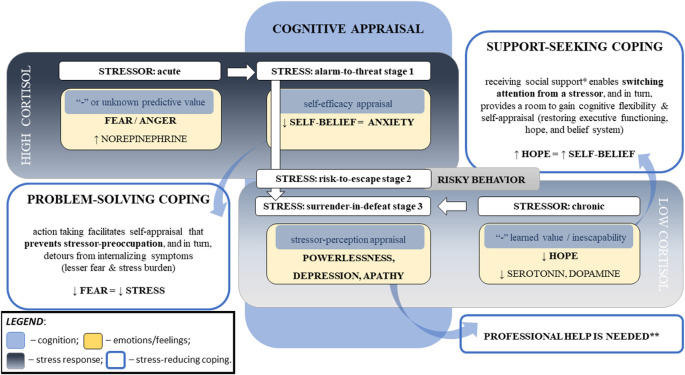
Major cognitive determinants of the cortisol responses linked to stress psychopathology. Note. This diagram represents the major factors influencing cortisol response to stress that can lead to stress disorders. Stress responses depend on the particular challenge, one’s perception of the stressor, and the ability to cope with the stressor. The stressor’s intensity, acuity, and persistence relate to cortisol responses, which are moderated by cognitive appraisal that is associated with self-efficacy and coping abilities. The stressor’s novelty (i.e., unknown predictive value) and inescapability (i.e., negative “learned” value) increase negative predictive values (i.e., fear and powerlessness, respectively), that hinder self-appraisal and aggravate stress severity. Repeated exposure to homotypic stressors resets the hypothalamic–pituitary–adrenal axis. Chronic stress can result in blunted cortisol responses to a stressor, flattened diurnal slops, and increased cortisol awakening responses. *Legend*: *—not limited to the emotional aspect that reduces stress perception (e.g., motivation, compassion^1^, and sense of belonging^2–4^) but also social and physical aspects directed to a reduction in the stressor’s influence (e.g., physical or financial help); **—risk of PTSD and suicidal ideation; ↑: increase; ↓: decrease, “-”: negative; ^1^Vaillancourt and Palamarchuk (2021); ^2^Grobecker (2016); ^3^Choenarom et al. (2005); ^4^Stachl and Baranger (2020).

Other findings support the impact of self-appraisal on stress severity. In adolescents, Sladek et al. (2016) showed that higher levels of perceived daily stress severity were linked to elevated cortisol levels, compared to diurnal patterning, only in: (1) individuals with low self-appraisal; and (2) in situations with higher “engagement” coping (i.e., support seeking). The situational variation of cortisol reactivity likely indicates that engagement coping may be due to lower self-belief in coping capacity and thus lower self-appraisal. Coping efficacy related to self-belief in one’s capacity to deal with a stressful situation has been found to be linked to psychological problems in children of divorced parents (Sandler et al., 2000). In another study, compared to peers with high coping efficacy, adolescents with increased loneliness and low coping efficacy presented a flatter diurnal cortisol slopes, a marker of poor cortisol regulation, later on in college; while higher coping efficacy predicted lower levels of the cortisol awakening response in college (Drake et al., 2016). In their subsequent work, Sladek et al. (2017b) found that girls with an active engagement coping style in response to interpersonal stress had lower cortisol levels (measured by diurnal cortisol slope, total output across the day (AUCg), and cortisol awakening response). However, higher rates of using active coping related to higher cortisol awakening responses the next morning. For women with attentional avoidance of social threat cues, Sladek et al. (2017a) showed that increased use of social support coping predicted lower cortisol responses to social stress and flatter average diurnal cortisol slopes compared to women with attentional vigilance (i.e., a bias toward threat). Similar cortisol patterns were found in children who had more social problems compared to their peers, which was seen in flatter slopes of cortisol decline from wakening to bedtime; as well, children presented with higher cortisol at wakeup time the next morning after higher than usual rates of peer or academic problems at school (Bai et al., 2017; see Figure 3).

The impact of self-appraisal on stress response/severity is in keeping with meta-analytic results by Kammeyer-Mueller et al. (2009), which demonstrated that core self-evaluations (i.e., a stable personality trait that encompasses self-efficacy, locus of control, self-esteem, and neurotism) related to lower perceived stress, higher rates of problem-solving coping, reduced strain, and lower levels of engagement in avoidance coping. In this meta-analysis, self-appraisal was not significantly linked to emotion-focused coping and emotional stability moderated the association between stress and strain and was uniquely linked to the coping process and stress. A meta-analysis by Connor-Smith and Flachsbart (2007) adds to the idea that personality traits can predict higher rates of specific coping strategies, including problem-solving and cognitive restructuring (for extraversion and conscientiousness), support seeking (for extraversion), and wishful thinking (i.e., mental avoidance), withdrawal, and emotion-focused coping (for neuroticism).

The effect of self-appraisal may be related to the aforementioned sensory-driven shift in the LC firing in response to stress, that suppresses goal-orientated actions, which need to be balanced with the action-orientated switch (i.e., subconsciousness “cognitive defence task”). In other words, sufficient self-appraisal supports self-belief and reduces the “mental barriers”, which in turn facilitates active, problem-solving coping. Further research is needed to lend more clarity on these associations (see Figure 2). A meta-analysis by Penley et al. (2002) showed problem-solving coping, but not emotion-focused coping, was associated with positive outcomes on general physical and psychological health. The nuances were that deliberate actions or analytical efforts and problem-focused coping were helpful only in acute interpersonal stress, correlating positively to psychological health outcomes. The effect was opposite in chronic stress, correlating negatively to psychological health outcomes. This highlights the fact that chronically distressed individuals do require social/psychological assistance. In contrast, seeking social support, confrontation, self-blame, mental or physical avoidance/distancing, self-control, and positive reappraisal in which emphasis is placed on a positive side of a situation, correlated with poor psychological self-reported outcomes in acute stress.

The major role of self-appraisal aligns with Social Self Preservation Theory (Gruenewald et al., 2004; see also Dickerson and Kemeny, 2004). For instance, in social evaluative stress, both acceptance threat and status threat can elicit a cortisol response (Smith and Jordan, 2015), and threats to the social self can induce shame and reduce self-esteem, which correlates with stress-induced cortisol levels (Gruenewald et al., 2004). It has also been demonstrated that high cortisol in social evaluative stress is accompanied by sympathetic activation (i.e., hyperarousal due to the NE surges), but not parasympathetic activation (i.e., measured by heart rate variability, can relate to affective responses; Bosch et al., 2009; Mackersie and Kearney, 2017; Poppelaars et al., 2019). Further, the magnitude of the stress response has been shown to increase in women with the size of the audience (Bosch et al., 2009), whereas sympathetic hyperreactivity was found to predict increased reactivity of the hypothalamic–pituitary–adrenal (HPA) axis, again in women (Poppelaars et al., 2019).

Stress perception also moderates the impact of a stressor on neurocognitive function. For instance, Jiang et al. (2017) showed that higher levels of stress perception correlated with poor episodic memory and frontal executive function in older adults free of mild cognitive impairment and dementia. Higher stress severity can be experienced in novel/unpredictable and inescapable conditions (e.g., Sauro et al., 2003; Lupien et al., 2007; Slattery et al., 2013) and is distinguished by hyperarousal. Tsuda et al.’s (1989) rodent studies, where these types of conditions, but not predictable stress, elevated NE in the LC and corticosterone in plasma. The apparent effect of the compromised feeling of control over unknown/novel challenges or in learned helplessness, aligns well with the self-appraisal influence discussed above. Dickerson et al.’s (2004) meta-analysis provides evidence that uncontrollable social threat relates to the highest levels of cortisol and adrenocorticotropin hormone responses to stress and the longest post-stress recovery.

Aversive emotions in both stress and stress anticipation that result in NE surge affect cortisol influence on attention, cognitive flexibility, memory, and learning, and thus aggravate the intensity of a stressor (Skosnik et al., 2000; Morilak et al., 2005; Alexander et al., 2007; Kvetnansky et al., 2009; Gray et al., 2017). That is, in intense stress, negative emotions enhance aversive memories and withdraw the cognitive focus from the “peripheral” details. Such selective attention is associated with poor working memory and memory retrieval (de Quervain et al., 1998, 2009; Roozendaal et al., 2006, 2008). The effect of emotional valence in stress involves concurrent activation of glucocorticoid receptors (GRs) and adrenoreceptors, specifically, central β-adrenergic receptors activation linked to long-term declarative memory for emotionally arousing information (e.g., Cahill et al., 1994; Cahill et al., 2004; Maheu et al., 2005a, b; see also Gibbs and Summers, 2000, 2002; Schwabe et al., 2009; Smeets et al., 2009; Lonergan et al., 2013) and activation of α_1_-adrenoreceptors that were insensitive previously to NE in the medial entorhinal cortex, linked to hippocampal memory dysregulation (e.g., Carrion and Wong, 2012; Hartner and Schrader, 2018). As well, a deletion variant gene that encodes α_2B_ adrenoceptor, ADRA2B, contributes to the cognitive processing of emotional information (see meta-analytic review by Xie et al., 2018). Levels of hyperarousal and its proximity to the occurrence of stress modulate memory formation, whereas higher hyperarousal can be seen in children due to neurodevelopmental sensitivity (e.g., Palamarchuk and Vaillancourt, under review; Vaillancourt and Palamarchuk, 2021), and in women due to the LC-NE system specifics (e.g., Bangasser et al., 2016; Bangasser and Wicks, 2017; Bangasser et al., 2018, 2019; see also Mulvey et al., 2018). Additionally, the sex differences are that emotionally influenced memory relates to hyperactivated amygdala with a stronger effect in the left hemisphere for women and in the right hemisphere for men (e.g., Cahill et al., 2004). Animal studies on fear conditioning show that mild-to-low levels of hyperarousal can impair spatial recognition memory, yet moderate-to-strong levels of hyperarousal can enhance the memory (e.g., Baars and Gage, 2010; Conrad, 2010). Therefore, stress reactivity has inter-individual variations that can be mild or more pronounced depending upon the individual’s stress appraisal and valence of aversive emotions, which are moderated by age and gender. Additionally, glucocorticoid stimulation followed hours earlier by NE secretion has been shown to inhibit arousal effect on memory (Osborne et al., 2015).

## Decision Making and Stress

The executive functioning facilitates adaptation with decision-making based on the evaluated external (environmental) and internal (sensory) information (e.g., De Kloet et al., 1998; Wager and Smith, 2003; Collins and Koechlin, 2012; Barbey et al., 2013; Dajani and Uddin, 2015). Executive functioning integrates memory, cognitive flexibility (such as rapid attention and task-shifting, as well behavioral adjustments, e.g., Palamarchuk and Vaillancourt, under review), learning fortification, reasoning, insecurity predictability, and monitoring behavioral strategies (e.g., Collins and Koechlin, 2012; see also Grissom and Reyes, 2019). The distinctions are that the ventromedial PFC integrates memory and emotional systems that are needed for decision-making, whereas the striatal and ACC inputs can affect it with bias (e.g., Gupta et al., 2011; Ho et al., 2012; Shimp et al., 2015; Goulet-Kennedy et al., 2016; Fitoussi et al., 2018; Hiser and Koenigs, 2018; Palamarchuk and Vaillancourt, under review). At the same time, the amygdala mediates emotional responses that engage the insula, which relates to social pain, empathy, and anger (e.g., Palamarchuk and Vaillancourt, under review). In a social context, the medial PFC and amygdala, but not ventral striatum, moderate decision-making (Ho et al., 2012; see also Hiser and Koenigs, 2018); whereas high levels of fear or anger (i.e., the amygdalar hyper response to a stressor) can affect decision-making with impulsivity/immediate actions (e.g., Gupta et al., 2011). Conversely, the stress associated with uncertainty and unknown power over a situation involves the frontrostriatal circuits, where task-sets and actions are driven by the references of cognitive/behavioral strategies stored in the long–term memory as a *script* (relates to the dorsal striatum/left caudate nucleus engaged in reward and motivation). Thus, in the context of stress-related ambiguity, the choice depends on predicted outcome values (related to the ventral striatum/the nucleus accumbens and ventral putamen engaged in cognitive control) to maximize their utilization, i.e., reinforcement learning/instrumental conditioning (O’Doherty et al., 2004; see also Hollerman et al., 2000; Brovelli et al., 2011; Vogel et al., 2015, 2017). The strategy is selected if it is absolutely reliable (the ventral striatum, nucleus accumbens) among the assortment of scripts (the dorsal striatum, nucleus caudate); and if it is unavailable, a new task-set is created because the decision-making is binary when the stimulus is ambiguous (e.g., Collins and Koechlin, 2012).

Emotional state/mood can affect the interpretation of the stressor, i.e., the mood-incongruent effects. Anxiety can lead to attentional bias toward threat due to higher predicted negative outcome of the stressor (i.e., ambiguity (fear, e.g., Blanchette and Richards, 2003; Barazzone and Davey, 2009). An anxious state also increases speed in the detection of aversive changes on a subliminal level and increases attention and conscious awareness on a supraliminal level (Gregory and Lambert, 2012). For example, in adults with high trait anxiety, the anxious state lowers awareness thresholds. In particular, fearful faces or non-threat faces presented among threatening faces are detected faster (Ruderman and Lamy, 2012). Neurocognitive functioning in stress thus drops cognitive flexibility (i.e., reduced functions of the dorsolateral PFC) to stay focused on the stressors, this attentional tunneling during emotional arousal allows the individual to detach from the “peripheral” information unrelated to the stressor that might distract the individual who is under pressure (e.g., Palamarchuk and Vaillancourt, under review; see also Brosch et al., 2013; LeBlanc et al., 2015). However, attentional tunneling and enhanced memory for aversive experiences can lead to psychological maladjustment, for instance, emotion-focused coping, anxiety, and PTSD (e.g., Palamarchuk and Vaillancourt, under review).

## Discussion

### Hypothesis: Coping Mechanisms Are Driven by the Stress Stages

We define coping styles as intra-individual neurocognitive variability moderated by stress development across three main stages: (1) *alarm-to-threat stage* → (2) * risk-to-escape stage* → (3) *surrender-in-defeat stage.* Potentially, the full development can be observed in chronic, intense, and homotypic stress associated with the HPA resetting and circulating cortisol decline. It is likely that these stress stages can be disrupted/attenuated, escalated, and/or distorted according to the level of perceived stress severity and neuropsychological status; whereas novel stressors can restart stress phases cycling (e.g., stress detection phase I; see Figure 1). Therefore, coping styles can fluctuate in a predictable intra-individual manner and recognizing the stress stage can expedite adequate interventions to prevent or treat maladaptive coping.

### Alarm-to-Threat (Check) Stage

Acute intense stress triggers right amygdalar fear-related effects such as tunneling attention, anxiety, and impulsivity seen in a reactive aggression as a sympathetic *fight-or-flight* response that is driven by high cortisol and NE levels (e.g., Palamarchuk and Vaillancourt, under review). The core mechanism is that fear can initially serve adaptation by reducing risky behavior (e.g., Pabst et al., 2013a, b; Yu, 2016; Vogel and Schwabe, 2019), because, in contrast, positive emotions can increase the probability of risk-taking (e.g., LeBlanc et al., 2015). Specifically, aversive emotions during mild psychological stress can facilitate the most reliable cognitive strategy *via* the narrowed scope of attention (that can also be induced by the pre-goal desire, e.g., LeBlanc et al., 2015), reduced configural associative learning (i.e., reduction in tri-/biconditional discrimination), and enhanced binary (uniconditional as irrelevant vs relevant) discrimination (e.g., Byrom and Murphy, 2016). Of relevance, social stress has been shown to increase activity in the anterior PFC associated with parallel processing during decision-making performance (e.g., the Game of Dice Task, Gathmann et al., 2014; see also Schiebener and Brand, 2015; Shimp et al., 2015). However, stimuli associated with extreme/traumatic experiences can trigger inadequate responses and reduce responses to contextual cues such as focusing on aversive sound and disregarding the safety of the environment that promotes automatic retrieval of traumatic experiences (e.g., Cohen et al., 2009; Otgaar et al., 2017). This is an example of accentuated alarm-to-threat stage by rigid binary cognitive strategy, whereas improving cognitive flexibility by configural associative learning could be a key element in the psychotherapeutical approach. Another example is that strong fear can elicit avoidance behavior related to the left lateral amygdala and anterior hippocampal hyperactivity (Abivardi et al., 2020). In other words, “cold” executive functioning is set to prioritize the most reliable decision-making to avoid danger when confronting a threat, yet it limits attention and flexibility. The mechanism is facilitated by promoted dorsal striatum-dependent (“habit”) learning and behavior over hippocampal-dependent (“cognitive”) memory encoding and retrieval, which leads to stereotypical ideas and thus maladaptive functioning in chronic stress (e.g., Packard, 2009; Vogel and Schwabe, 2016; Vogel et al., 2017; Zerbes et al., 2020; see also Schiebener and Brand, 2015; Shimp et al., 2015; Fitoussi et al., 2018). In particular, poor consequences can be seen in attentional set-shifting deficits, poor memory, anxiety, and depression (e.g., Palamarchuk and Vaillancourt, under review).

If acute stress subsides, attention can be improved with the decline of cortisol (e.g., Zandara et al., 2016). Conversely, intense stress can hyperactivate the LC that is associated with anxiety (Borodovitsyna et al., 2018; Morris et al., 2020) due to limbic dysregulation (e.g., Herman et al., 2005). In particular, it is related to the functional connectivity between the bed nucleus of the stria terminalis (BNST) and amygdala (e.g., Clauss, 2019; Knight and Depue, 2019; Hofmann and Straube, 2021). The nuances are that the amygdala is involved in explicit threat processing (i.e., threat confrontation), whereas the BNST is involved in ambiguous threat processing (i.e., threat anticipation; Herrmann et al., 2016; Klumpers et al., 2017; Naaz et al., 2019; see also Fox et al., 2015; Fox and Shackman, 2019; Luyck et al., 2019). As well, the BNST → central amygdala projections relate to cued-fear inhibition (Gungor et al., 2015; see also Clauss, 2019). The BNST plays a critical role in fear acquisition/expression, which relates to stress maladaptation and the development of stress-related disorders like PTSD (e.g., Miles and Maren, 2019) and involves CRH signaling (e.g., Hu et al., 2020). This functional interplay between the BNST and amygdala relates to the inter-individual differences in threat processing and trait anxiety (Brinkmann et al., 2018), which likely influences the development of the next stage in chronic intense stress.

### Risk-to-Escape (Stalemate) Stage

The evidence is that stress, predominantly chronic, can increase risk-taking behavior (Starcke et al., 2008; Lighthall et al., 2009; Pabst et al., 2013c; Ceccato et al., 2016; see also Brand et al., 2006; Starcke and Brand, 2012; Yu, 2016). We predict that stress-induced risk-taking is largely driven by threat anticipation due to hyperactivated BNST. The BNST integrates limbic information and valence monitoring and plays a central role in the hippocampus-hypothalamic paraventricular nucleus circuit that activates the HPA axis and has a psychogenic effect (e.g., Lebow and Chen, 2016). The BNST is sexually dimorphic; its activity is heritable and relates to anxiety in ambiguous and sustained threat (e.g., Clauss, 2019). The neurophysiological background is that the BNST receives multiple signals, including, but not limited to, dopamine and 5-HT from the dorsal raphe and NE from the nucleus tractus solitarii (e.g., Glangetas and Georges, 2016). Moreover, increased impulsivity relates to alteration in the central amygdala → BNST dopaminergic projections that inhibit impulsive behavior (Kim et al., 2018).

We thus predict that in prolonged homotypic stress, hyperactivated BNST *covers a shift from the front-line stress-care* medial PFC-amygdalar circuits. This is likely a *now-or-never* response to escape the burden of anticipated threat, driven by dopamine reductions in uncertain conditions which recruit the dorsal PFC-striatal circuits related to impulsive and risky behavior. Our reasoning is that, in contrast to fear, ambiguity can be perceived as a *dormant threat* that increases approach behavior (the hippocampal rectivity, e.g., O’Neil et al., 2015) and risky behavior (the ventral striatal reactivity moderated by impulsivity traits, e.g., Mason et al., 2014; Goulet-Kennedy et al., 2016). As well, the activity of the ventral striatum is associated with a motivational control of performance and is regulated by the dorsolateral PFC (Hart et al., 2014). Therefore, it could be a part of an adaptive mechanism to confront the challenge although it requires adequate executive functioning, and by extension, goal-oriented actions. The pitfalls are that poor cognitive control and insular risk-processing can increase perceived stress, and in turn, risk-taking behavior (e.g., among adolescents, Maciejewski et al., 2018). In contarst, risk-taking behavior is inversely associated with a cortisol increase for boys/men but not girls/women (e.g., Daughters et al., 2013; Kluen et al., 2017). This effect relates to greater activity and novelty preferences due to higher sensation seeking in boys/men compared to girls/women who are more punishment sensitive (meta-analysis by Cross et al., 2011). The developmental moderation of stress-induced responses can also lead to impulsive errors in girls (e.g., Lukkes et al., 2016), which is also moderated by personality traits related to impulsivity (e.g., negative urgency that correlates to impulsivity, Berg et al., 2015; see also Cyders and Smith, 2008a, b; Herman et al., 2018). The levels of impulsivity in healthy young adults inversely correlate with the levels of released dopamine from the ventral striatum in low to moderate stress; yet high stress reduces dopamine responses (e.g., Oswald et al., 2007; see also Palamarchuk and Vaillancourt, under review).

In sum, poor cognitive functioning and cortisol decline can promote a burden of uncertainty (*stalemate*), and as dopamine drops, risk-taking ensues to which young men are more prone to than young women. The mechanism is that the striatal networks can serve decision-making with the learned behavior/”script” when facing explicit danger in acute stress. In contrast, when dealing with prolonged uncertainty, decision-making can be impulsive and risky due to poor risk-processing, and potentially, motivation/urge to terminate the *status quo* in chronic intense stress. Accordingly, improving cognitive control with proper risk-processing (psychological help) and facilitating adequate options to avoid predictable danger (social assistance) could be a key intervention to prevent poor outcomes. Although our hypothesis has yet to be tested, it sheds light on why stress can induce risk-taking behavior.

### Surrender-in-Defeat (Checkmate) Stage

We interpret that in acute and extreme stress associated with a loss or defeat, as well as in chronic stress with a prolonged ambiguity, the executive functioning “surrenders” in the absence of absolutely reliable task-sets and incapacity to create new ones (i.e., defeat/*checkmate*), which is why serotonin levels drop and depression emerges. Of relevance, Yu et al.’s (2016) findings in rodent models demonstrate that repeated social defeats, but not social threats, increase cortisol and NE levels but decrease dopamine, its metabolites, and serotonin levels in the striatum and hippocampus (see also Palamarchuk and Vaillancourt, under review).

On a molecular level, stress adaptation relates to a negative feedback of the HPA axis seen in cortisol hyposynthesis as ACTH sensitivity declines (e.g., Juruena et al., 2003; McEwen, 2012; Gray et al., 2017). In particular, the duration of exposure to a homotypic stressor displays a linear and inverted U-shaped dose-effect on a stress response: (1) a novel stressor can increase ACTH sensitivity; (2) a repeated stressor can initially desensitize ACTH; and (3) a chronic stressor relates to an unceasing ACTH sensitivity (Aguilera, 1994, 1998; Aguilera and Liu, 2012). Prior exposure to homotypic stressors can compromise stress response to a novel stressor (e.g., García et al., 2000), which in turn can expose a previous stress-induced latent behavioral sensitization that often surpasses the HPA axis sensitization (Belda et al., 2015; also see McCarty, 2016). Not surprisingly, intense stressor can facilitate certain cognitive functions and thus promote stress resilience (e.g., Ellis et al., 2017) although its chronic exposure is associated with mood disorders such as depression and anxiety (e.g., Juruena et al., 2020). According to the aforementioned findings on stress responses, we hypothesize that intra-stages expressions and inter-stage transitions in our model of stress development depend on the novelty, intensity, timing, and chronicity of the stressor. Stress stages can be desensitized in subchronic exposure to the same stressor (or homotypic stressors) but accelerated/exacerbated in chronic exposure to the homotypic stressors, which in turn can also hypersensitize stages toward a novel stressor.

We acknowledge that sex/gender may affect the coping-related neural pathways due to sex and stress hormones co-signaling. In particular, neurocognitive variability during stress development can be affected by the levels of circulating estradiol/estrogen. Estrogen signaling influences memory, social learning, and aggressive/defensive behavior associated with the hippocampal and medial PFC functioning (e.g., Milner et al., 2008; Luine and Frankfurt, 2012; Laredo et al., 2014; Almey et al., 2015) and thus contributes to sex differences in stress coping. In females, circulating estradiol levels mediate stress resilience (e.g., Wei et al., 2014b; Luine, 2016; Yuen et al., 2016) and facilitate cerebro- and cardio-protection (e.g., Guo et al., 2005; Murphy, 2011; Adlanmerini et al., 2014) in linear and inverted U-shaped dose-effect (e.g., Bayer et al., 2018), where high estrogen levels increase cognitive sensitivity to stress (e.g., Graham and Scott, 2018; Hokenson et al., 2021). On the one hand, this may help explain why the prevalence of PTSD—*surrender-in-defeat stage* in our model—is two times higher in women than in men (e.g., Breslau, 2002; Zlotnick et al., 2006; Pooley et al., 2018). On the other hand, the androgen effect may explain the findings of why men are inclined toward impulsive behavior (i.e., *risk-to-escape stage* in our model, e.g., Hernandez et al., 2020) and are more affected by stress magnitude, compared to women who are more affected by stress frequency (e.g., Grissom and Reyes, 2019; see also Hidalgo et al., 2019).

Our hypotheses need to be tested to further clarify the various interfering factors with stress reactivity and resilience, such as sex hormones and genetic polymorphism related to serotonin and dopamine signaling reviewed above, as well as stressor type and stress timing/continuity (single, repeated intermittent, or chronic) that can involve different neural pathways and different reactivity of the HPA axis and LC-NE system. Nevertheless, these hypotheses can help explain why active coping is negatively linked to psychological health as reviewed above (Figure 1). It also supports the fact that chronically stressed individuals with depression/anxiety and poor cognition require psychological and social assistance.

### Concluding Remarks

Neurocognition plays a vital role in adaptation and monitors the severity of challenges faced. When cognitive appraisal assigns a negative value to the salient stimuli, it is the moment they become psychological stressors and stress arises. Thus, psychological stimuli can vary in nature because it is the level of cognitive “attention” that determines stress and its severity, that is the stress appraisal/interpretation, but not the stimuli *per se*.

To address the nuances underlining stress severity, we propose to update a dichotomy in the cognitive appraisal terminology—*self-appraisal* (i.e., the perception of self-efficacy to deal with the stressor) and *stressor-appraisal* (the perception of threat/challenge). This dichotomy is intended to facilitate cognitive behavioral therapy, as well as translational research on stress and mental resilience. Specifically, *self-appraisal* relates to successful emotional downregulation and enables cognitive flexibility vs. *stressor-appraisal* which can contribute to emotional dysregulation and attentional tunneling that restricts/alters executive functioning. Noted specifics of the cognitive appraisal duality are associated with the PFC and amygdala interplay during the processing of aversive emotions and fear, which is linked to stress sensitization and psychiatric consequences (e.g., Palamarchuk and Vaillancourt, under review).

To advance our understanding of mental resilience and stress development, we offer new insights to the scholarly literature on psychological stress coping with respect to previously published reviews. First, we differentiate the neurocognitive aspects in stress development with four key phases: (i) stressor detection, (ii) stress appraisal (assessment of stress severity), (iii) stress reactivity, and (iv) decision making. Clinical analysis of each phase may help with ruling out primary and secondary causes of behavioral maladaptation. For instance, it is important to keep in mind that sudden and inadequate behavioral reaction to an event (i.e., detection of a novel stressor) may be related to a totally different event that occurred chronically in the past that latently compromised psychological health (i.e., prior chronic exposure to homotypic stressors can trigger cognitive “defence,” see Figure 1). Another example is that prolonged uncertainty increases the chances of risky/impulsive behavior.

Second, we model a complex concept of stress development that introduces an intra-individual variability factor in the stress reactivity phase, which is based on the neural dynamics in cognitive processing. In particular, we hypothesize that coping styles are influenced by intra-individual neurocognitive variability moderated by stress reactivity (phase iii) across three major stages: (1) alarm-to-threat [check] stage → (2) risk-to-escape [stalemate] stage → (3) surrender-in-defeat [checkmate] stage (Figure 1). *Alarm-to-threat stage* denoting the cortisol and NE surges in response to psychological stress must not be confused with the *alarm phase*, classically referred to triphasic allostasis process, which originated from the “general adaptation syndrome” concept by Selye (1998), reprint of 1936) that described “typical syndrome” following “diverse nocuous agents.” That is, the general alarm reaction within “6–48 h in rat models of acute nonspecific stress.”

Finally, we emphasize that stress coping can fluctuate in a predictable intra-individual manner. Identifying the stressor’s novelty/chronicity and stress stage/phase can help with early prevention and appropriate therapy of maladaptive stress coping, and in turn, prevent mental disorders.

## Author Contributions

TV encouraged, supported, and supervised ISP to investigate stress impact on cognition. ISP planned and carried out the project, the main conceptual ideas, developed the theoretical models and hypotheses, and designed the figures. ISP wrote the manuscript with support from TV. ISP and TV provided critical feedback, helped shape the manuscript, and contributed to the final version. The authors are accountable for the content of the work. All authors contributed to the article and approved the submitted version.

## Conflict of Interest

The authors declare that the research was conducted in the absence of any commercial or financial relationships that could be construed as a potential conflict of interest.

## Publisher’s Note

All claims expressed in this article are solely those of the authors and do not necessarily represent those of their affiliated organizations, or those of the publisher, the editors and the reviewers. Any product that may be evaluated in this article, or claim that may be made by its manufacturer, is not guaranteed or endorsed by the publisher.
